# Scoping Review of National Antimicrobial Stewardship Activities in Eight African Countries and Adaptable Recommendations

**DOI:** 10.3390/antibiotics11091149

**Published:** 2022-08-25

**Authors:** Nduta Kamere, Sandra Tafadzwa Garwe, Oluwatosin Olugbenga Akinwotu, Chloe Tuck, Eva M. Krockow, Sara Yadav, Agbaje Ganiyu Olawale, Ayobami Hassan Diyaolu, Derick Munkombwe, Eric Muringu, Eva Prosper Muro, Felix Kaminyoghe, Hameedat Taiye Ayotunde, Love Omoniyei, Mashood Oluku Lawal, Shuwary Hughric Adekule Barlatt, Tumaini J. Makole, Winnie Nambatya, Yvonne Esseku, Victoria Rutter, Diane Ashiru-Oredope

**Affiliations:** 1 Commonwealth Partnerships Programme on Antimicrobial Stewardship, Commonwealth Pharmacists Association, London E1W 1AW, UK; 2 Department of Neuroscience, Psychology and Behaviour, University of Leicester, Leicester LE1 7RH, UK; 3 Pharmacy Department, University of Zambia, Lusaka P.O. Box 50110, Zambia; 4 Projects Department, Pharmaceutical Society of Kenya, Nairobi P.O. Box 44290-00100, Kenya; 5 Department of Pharmacology, Kilimanjaro Christian Medical University, Kilimanjaro P.O. Box 2240, Tanzania; 6 Pharmaceutical Society of Malawi, Lilongwe P.O. Box 2240, Malawi; 7 Pharmaceutical Society of Nigeria, Lagos P.O. Box 531, Nigeria; 8 Drug Information Services & Quality Assurance Unit, Directorate of Pharmaceutical Services, Freetown P.O. Box 232, Sierra Leone; 9 Pharmacy Department, College of Health Sciences, Makerere University, Kampala P.O. Box 7062, Uganda; 10 Ghana College of Pharmacists, Accra P.O. Box CT 10740, Ghana

**Keywords:** antibiotic resistance, CwPAMS, national action plans, pharmacy, One Health

## Abstract

Antimicrobial resistance (AMR) is a global health problem threatening safe, effective healthcare delivery in all countries and settings. The ability of microorganisms to become resistant to the effects of antimicrobials is an inevitable evolutionary process. The misuse and overuse of antimicrobial agents have increased the importance of a global focus on antimicrobial stewardship (AMS). This review provides insight into the current AMS landscape and identifies contemporary actors and initiatives related to AMS projects in eight African countries (Ghana, Kenya, Malawi, Nigeria, Sierra Leone, Tanzania, Uganda, and Zambia), which form a network of countries participating in the Commonwealth Partnerships for Antimicrobial Stewardship (CwPAMS) programme. We focus on common themes across the eight countries, including the current status of AMR, infection prevention and control, AMR implementation strategies, AMS, antimicrobial surveillance, antimicrobial use, antimicrobial consumption surveillance, a one health approach, digital health, pre-service and in-service AMR and AMS training, access to and supply of medicines, and the impact of COVID-19. Recommendations suitable for adaptation are presented, including the development of a national AMS strategy and incorporation of AMS in pharmacists’ and other healthcare professionals’ curricula for pre-service and in-service training.

## 1. Background

Antimicrobial resistance (AMR) is a worldwide public health threat of global dimensions. The 2016 Review on AMR commissioned by the UK Government estimated that, by 2050, antimicrobial-resistant infections could be responsible for 10 million deaths globally each year. This is more than cancer, road traffic accidents, diabetes, and diarrhoeal diseases, with developing countries and large emerging nations bearing the brunt of this problem [1]. The impact of this will be felt in all healthcare provisions, with routine surgeries and minor infections becoming life-threatening once again. In 2019, it was estimated that the highest rates of AMR burden were in sub-Saharan Africa, among all regions [2]. However, in sub-Saharan Africa, inadequate surveillance and regulation make it challenging to define the accurate status of AMR and to implement effective and sustainable programmes to address it [3].

According to country-specific situational analyses, joint external evaluation reports, and other studies from hospital to the national level, worsening AMR trends have been identified in many low- and middle-income countries (LMICs) within the African continent. Coupled with the burden of infectious diseases, such as tuberculosis (TB), respiratory infections, and diarrhoeal diseases, emerging resistance to treatments for these diseases has a potentially devastating impact on healthcare in these countries [4,5]. AMR has been exacerbated in these contexts by the irrational use of medicines and national guidelines have identified the need to address inadequate AMR surveillance and enable legislation to monitor the rise in AMR [6,7].

This paper presents findings from a rapid scoping review to assess national antimicrobial stewardship (AMS) activities (including those following a One Health approach) in eight African countries (Ghana, Kenya, Malawi, Nigeria, Sierra Leone, Tanzania, Uganda and Zambia) that were carried out as part of the Commonwealth Partnerships for Antimicrobial Stewardship (CwPAMS) programme [8]. CwPAMS provided the useful setting for the review of existing AMS activities because the programme had established an infrastructure that enabled the easy identification of relevant national stakeholders to be included in the scoping process. Given the CwPAMS partnerships’ efforts in running AMS interventions in LMICs, the project further provided the ideal context for assessing existing AMS and infection prevention and control (IPC) structures (including the One Health approach), as well as national policies and guidelines in place.

This review addresses three main research questions in relation to the eight African CwPAMS countries:

1. What is the current status of AMR?
2. What programmes of AMS and IPC are currently in place for human and animal health?
3. What national policies and guidelines exist to support these programmes?

By addressing these questions, the review aims to critically review and compare existing AMS approaches in sub-Saharan Africa and outline gaps or areas for improvement. We conclude by proposing a number of actionable recommendations to support ongoing efforts to tackle AMR in LMICs of the African continent and beyond.

## 2. Methods

**Figure 1:**
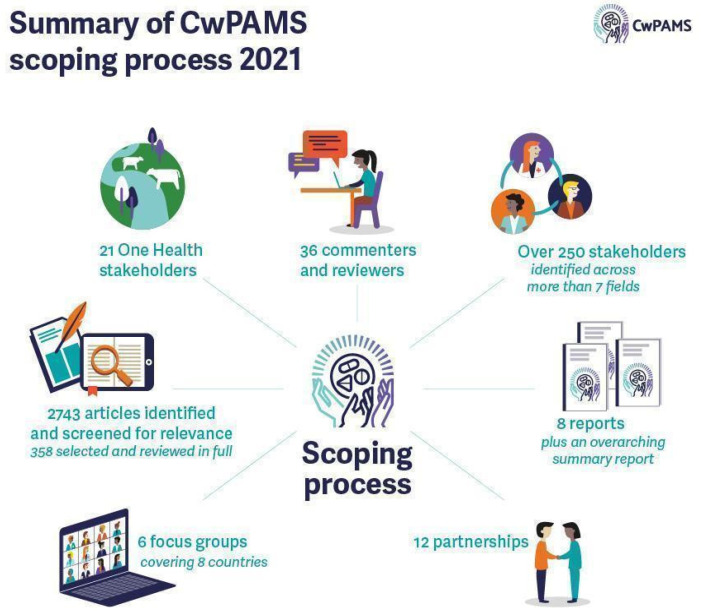
Summary of CwPAMS scoping process.

The CwPAMS scoping, Figure 1, involved a literature search using topics and keywords (detailed further in Box S1) relating to AMS conducted on the Medline database from 2015 to 2021 for each CwPAMS country. References to relevant articles were reviewed to identify any additional literature. This timeframe was chosen to ensure the timeliness and current relevance of the research. A total of 2197 articles were identified and screened for relevance against the scoping review objectives. A total of 267 articles were selected and reviewed in full. Citation and references were found in selected articles. An additional search using keywords was conducted for One Health literature for each country, resulting in 204 articles being identified and screened for relevance. Overall, 66 articles were included in the review [9,10,11,12,13,14,15,16,17,18,19,20,21,22,23,24,25,26,27,28,29,30,31,32,33,34,35,36,37,38,39,40,41,42,43,44,45,46,47,48,49,50,51,52,53,54,55,56,57,58,59,60,61,62,63,64,65,66,67,68,69,70,71,72,73,74]. With English being an official language in all eight African countries included in this review, only publications in English were screened.

Additionally, purposive searches of key organisational websites, such as respective government websites and World Health Organization, were conducted for reports and policy documents. This was supported by local expert correspondence to identify additional relevant literature from stakeholders and acting bodies in each country. This identified 342 relevant articles for the scoping review.

The study findings informed the discussion guide for a series of focus group discussions with participants and stakeholders in each country.

Between April and May 2021, workshops, key stakeholders and personal interviews, (Table S1), were conducted with a total of 55 experts with representation from National AMR Committees, Antimicrobial Stewardship, Ministries of Health, One Health, Animal and Environmental Health, Infection Prevention and Control, Medicine, Nursing and Pharmacy.

## 3. Findings

### 3.1. The Current Status of Antimicrobial Resistance

All the eight assessed countries previously participated in the WHO Joint External Evaluations (JEE), which informed the development of national action plans (NAPs) to implement international health regulations (IHR). A summary of recommended priority actions for AMR, which are currently under implementation, is explained further in the Box S2 [9,10,11,12,13].

Several studies in these countries have shown resistance to the most commonly used antimicrobials, such as penicillin, tetracyclines, ciprofloxacin, and cotrimoxazole [14]. Resistance to frequently used antimicrobials has been identified in multiple common microbial organisms, including *Staphylococcus aureus* and other Staphylococci (including Methicillin-resistant *Streptococcus pneumoniae*, *Salmonella typhi*, *Shigella* spp., *Escherichia coli*, and *Vibrio Cholerae* [6,7,15]. In addition, several studies suggest various drivers of AMR, mostly due to gaps in health systems’ capacity, supply, and regulation of antimicrobials across multiple sectors. These are detailed further in the Box S3 [7,15].

### 3.2. Antimicrobial Use, Monitoring, and Stewardship 

Evidence suggests inappropriate use of antimicrobials in hospitals, community settings, and veterinary treatment [10,11,12,13,16,17,18]. As a result, AMR prevention interventions have targeted healthcare facilities, communities, and the animal industry. There are variations in the status of AMS programme implementation in the eight countries and, despite recent strides, many face challenges, including leadership commitment to AMS, clinical pharmacy expertise, reporting, education, and accountability. Only one of the countries, Kenya, had specific national AMS guidelines for healthcare settings.

Country-specific studies at the hospital level further reported suboptimal compliance with treatment guidelines in healthcare-associated infections (HCAIs); lack of clarity in documentation in AMS; prolonged surgical antibiotic prophylaxis; extensive misuse of antibiotics (particularly in treating uncomplicated infections) and a lack of awareness among health workers.

### 3.3. Infection Prevention and Control (IPC)

IPC is a central precondition for reducing the need for antimicrobials. Our scoping search evidenced the existence of national IPC plans, policies, and documents to support IPC and water sanitation and hygiene (WASH) in healthcare facilities across the eight countries. However, IPC appears to be a neglected area at the healthcare facility level, and it is evident that there is a need for strengthening activities to address this.

There are little data on the incidence of (HCAIs), although it is a widely recognised priority issue to address. The actual burden of HCAIs in many LMICs is unknown due to microbiological data scarcity, inaccurate patient records, lack of electronic medical records, and inadequate surveillance systems to track HCAIs. However, studies indicate urinary tract infections (UTIs), surgical site infections (SSI), and healthcare-associated pneumonia (HAP) as common HCAIs in the eight African countries included in this review [19].

Effective IPC practices require training and monitoring of the health workforce. Barriers to IPC include a lack of adherence to training guidelines. Several studies were conducted on IPC knowledge, awareness, and practice by healthcare workers in the eight countries of interest. Results of the studies showed poor hygiene compliance due to inadequate training, poor infrastructure, and lack of personal protective equipment (PPE) in hospitals [20,21,22,23].

### 3.4. Use of WHO Tools and Participation in Assessments

Several WHO guidance and policy documents are available to inform national policy and decision-makers by providing data and guidelines on the burden of AMR, IPC and WASH, among other critical areas.

A range of WHO tools and guidelines used by the eight countries are summarised in Table 1.

These tools serve different purposes in providing guidance for the participating countries. Global antimicrobial resistance and use surveillance system (GLASS)-AMR provides a standardized approach to the collection, analysis, and sharing of AMR data by countries and seeks to support capacity development and monitor the status of existing or newly developed national AMR surveillance systems. The joint external evaluation (JEE) is a voluntary, collaborative, multisectoral process to comprehensively assess a country’s capacity to prevent, detect, and rapidly respond to public health risks in the framework of the international health regulations (IHR). The WHO point prevalence survey (PPS) methodology collects basic information from medical records and associated patient documentation on all hospitalized patients, which is of relevance for the treatment and the management of infectious diseases regardless of whether these patients are on antibiotic treatment at the time of data collection.

The national action plan (NAP) is a government document and is aligned with government policies, laws, regulations, and structures as necessary or relevant to AMR. Water and sanitation for health facility improvement tool (WASH FIT) is a risk-based approach for improving and sustaining water, sanitation and hygiene, and healthcare waste management infrastructure and services in healthcare facilities in LMICs. The global action plan (GAP) provides a framework for developing national action plans, including key actions that the various actors should take within 5–10 years to combat AMR. The tripartite AMR country self-assessment survey (TrACSS) is an annual evaluation jointly administered by the Food and Agriculture Organization (FAO), World Organisation for Animal Health (OIE), and WHO to monitor a country’s progress in the implementation of NAPs.

### 3.5. National AMR Strategies and Their Status of Implementation

National AMR action plans or strategies were available in all eight countries and included some or all of the necessary elements for effective AMS. These plans and strategies are led by an AMR coordinating committee (AMRCC) or a platform of multi-sector stakeholders who coordinate implementation activities. They represent a promising advancement in the form of increased policy awareness and commitment to tackle AMR. However, national AMR strategies varied in their degrees of implementation. Significant barriers remained, including gaps in operationalizing One Health interventions and a lack of monitoring processes for implementation. Additionally, input from in-country consultants indicated a lack of financial resources to effectively measure and establish the impact of AMR, e.g., through measuring cost-efficiency, quality-adjusted life years (QALYs), disability-adjusted life years (DALYs), mortality rates, costs associated with infectious disease, bed space, drugs, and treatment.

### 3.6. Notable Themes in National Action Plans

All eight countries adopted a multi-stakeholder approach to developing NAPs based on the country-specific Global Antibiotic Resistance Partnership (GARP) situation analysis recommendations. The stakeholders included WHO, FAO, Fleming Fund and respective government line ministries. Out of the eight assessed countries, the Malawi NAP was not readily available to stakeholders but was under review to improve implementation strategies.

Across all countries, representatives from different pharmaceutical bodies and pharmacists were involved in the drafting of the NAP. One of the NAP principles in all eight countries was using a One Health approach. There was a common understanding that AMR requires collective actions from all One Health components, including human and animal production, crop agriculture, and environmental activities.

Most of the countries’ NAPs contain clear implementation plans with monitoring and evaluation (M&E) indicators. However, there was limited documented evidence on the status of implementation of the NAPs. Fleming Fund country grant activities were implemented to support NAP development, along with an M&E plan to track progress in its implementation.

### 3.7. Surveillance of Antimicrobial Use (Including Point Prevalence Survey)

Most of the data that support treatment guidelines come from studies and surveillance systems from high-income countries (HICs). However, even in HICs, gaps still exist in the surveillance of bacterial pathogens that cause animal and human infections [24]. In addition, reports have shown that LMICs’ capacity for AMR laboratory testing is centralized, with minimal capacity at district level.

Several country-specific examples [24,25,26,27,28] are outlined in the Box S4.

### 3.8. Guidelines on Antimicrobial Use

The national clinical guidelines across all eight countries inform the prescription and dispensing of antimicrobials. However, Kenya was the only country out of the eight with dedicated national AMS guidelines for healthcare settings. In contrast, the rest of the countries have several updated guidelines, such as Standard Treatment Guidelines and Essential Medicines Lists, where AMS principles have been integrated. All countries have a broad legal framework for medicines control, including antimicrobials. However, there is currently limited literature on the implementation status of these guidelines.

### 3.9. Behavioural Barriers to AMS

Although policies on tackling AMR are available, they are often not implemented in practice due to resource limitations [29]. In addition, AMS activities are affected by the behaviour of health workers, individuals, and service providers. It was noted that structural barriers may result in additional behavioural barriers. Box 1 highlights behavioural barriers (including structural barriers) to tackling AMR in the eight countries [29,30,31,32,33]:

Box 1 **Behavioural barriers (including structural barriers) to tackling AMR [29,30,31,32,33].**

- Unrestricted access to antimicrobial medicines without prescription due to limited enforcement of legisla-tion;
- Individuals use antimicrobials to try to prevent infection, rather than using for treatment;
- Unaffordability of antimicrobials results in purchasing less than prescribed and patients tend not to finish their prescribed courses;
- Self-medication is common with medicines from market vendors, pharmacies, and drugs shared between friends and family;
- There is a lack of medical supplies, and long distances to health facilities;
- Poor attitudes of medical professionals towards patients;
- The misuse of antibiotics in hospitals, while not well documented, is evident, and there are no national guidelines for the use of antibiotics in intensive care unit (ICUs);
- Guidelines are not adhered to and hardly used, which may be due to a lack of faith in the advice;
- Up-to-date information on AMR is limited and not circulated or collated to be available from one source, so it does not influence clinical practice. This has led to the prescription of antibiotics being a matter of trial and error;
- Financial incentives exist through privately selling antibiotics in hospitals;
- Polypharmacy, including antimicrobials, and the use of fixed-dose combinations;
- Poor enforcement of laws and regulations on the handling of medicines. In addition, many drug shops and private clinics do not conform to the required standards and remain operational in many districts;
- Lack of diagnostic tests in medical decision-making, increasing or decreasing the probability of infection in a patient based on the result;
- Poor education on IPC and AMR;
- Characteristics of the facility and the healthcare worker, such as the healthcare worker’s knowledge and the availability of supplies, suggest that improvements will require a broader focus on behavioural change. Findings also emphasise the need to create hospital leadership buy-in in order to overcome challenges in effective AMS programme implementation, in the response to AMR.

### 3.10. Initiatives to Improve AMS

Focus group discussions highlighted the urgent need for progress with regard to AMS interventions. In addition to behavioural barriers to implementation, a lack of financial resources and competing national priorities often result in AMS work being side-lined.

Examples of current initiatives to improve AMS in the eight countries include:

- The African Institute for Development Policy (AFIDEP) developed awareness materials, such as comic strips and fact and information sheets, in the local Chichewa language in Malawi [34].
- The Drivers of Resistance in Uganda and Malawi (DRUM) consortium is a multi-stakeholder project working in Malawi and Uganda, researching AMR in humans, animals, and the environment [35].
- Nigeria utilises social media to promote AMS awareness and has an AMR Awareness Facebook page, where information is shared to support the implementation of the Global Action Plan on Antimicrobial Resistance and minimise the impact of AMR on human health, animal health, and the environment [36].
- A project was conducted to increase the awareness of AMR from February 2nd to May 13th, 2019, in Nigeria. The project used community outreach and social media to raise awareness about AMR and address issues, such as misconceptions and knowledge gaps. The student-led project had over 200,000 likes on Facebook, demonstrating how social media can be a powerful tool to reach the masses [37].
- In collaboration with relevant ministries, departments, and agencies in the animal and human health sectors, Nigeria’s Centre for Disease Control participates in World Antimicrobial Awareness Week activities every year. In 2020, under the theme ‘Antimicrobials: Handle with care’, several activities were conducted. These included a webinar regarding operationalising One Health interventions on AMS, engagement with livestock farmers, and training with Fleming Fund Fellows on AMR and antimicrobial use (AMU) surveillance [38].
- ReAct Africa assists countries, such as Zambia and Ghana, by bringing together experts and key stakeholders to form technical working groups on AMR. They ensure a multi-sectoral, holistic approach to AMR, targeting the general public, as well as the health, veterinarian, agricultural, and environmental sectors. Their role is to also increase collaboration with other relevant networks and organisations [39].
- In Kenya, both public and private hospitals, such as the Kenyatta National Hospital, Aga Khan University Hospital, and The Nairobi Hospital, have developed and implemented AMS programmes. They have observed significant adherence to AMU guidelines in surgical prophylaxis, restricted carbapenem, and other reserve antibiotic use, resulting in a decline in multi-drug resistant infections and candidemia. Other hospitals are in the process of establishing AMS teams with mentorship from hospitals that have implemented AMS programmes. Insights from the focus group discussions showed initiatives and unpublished documentation on AMS in the country. There is ongoing work with the United States Agency for International Development - Medicines, Technologies, and Pharmaceutical Services (MTaPS) Program (USAID-MTaPS), Fleming Fund, ReAct, CDC, WHO, FAO, and OIE.
- In 2017, Mbeya Zonal Referral Hospital (MZRH) and the University of South Carolina (UofSC) agreed on a collaborative project to strengthen antimicrobial prescribing in the southern highlands of Tanzania and train a new generation of clinicians in responsible AMU and adherence to national guidelines [40].

### 3.11. Guidelines on Infection Prevention and Control

Multiple partners, such as US CDC implementing partners, USAID-MTaPS, WHO, and ReAct, have been supporting countries to improve IPC. At the national level, in Kenya, they have provided technical assistance to the National Infection Prevention and Control Advisory Committee and the MoH Patient and Health Worker Safety Division to review the existing policies for health care workers. The National IPC Advisory and Coordination Committee supports and improves adherence to hand hygiene and waste management practices.

Several documents have been developed and implemented across all eight countries to facilitate IPC procedures and policies at the national and hospital level. They include but are not limited to: IPC plan; Infection Control and Waste Management plan; Standard Treatment guidelines; National Communication Strategy for IPC; National IPC Guidelines & Pocket Guides; Quality Improvement—IPC Orientation Guide for Participants; National IPC Standards; National Health Care Waste Management Policy Guidelines; HCWM Standards and Procedures; HCWM Monitoring Plan; National Catalogue for National HCWM Equipment and Facility Options; Essential Medicine List.

### 3.12. One Health Initiative in Relation to Antimicrobial Stewardship

#### 3.12.1. One Health Approach

Currently, in Ghana, Kenya, Nigeria, Tanzania, and Zambia, One Health collaboration is established within multi-sectoral working groups that operate using clear terms of reference and defined activities and follow arrangements pertaining to reporting and accountability [41]. By contrast, in Sierra Leone, One Health initiatives are established but did not have clear terms of reference and defined activities.

In Uganda, national strategic plans were reviewed by key stakeholders in the relevant ministries (Ministries of Health, Agriculture and Environment) to include the AMS/AMR agenda as part of their deliverables for further implementation. These efforts are expected to promote AMS activities at sub-national, district, and individual farmer levels. In Malawi, only a concept note exists on the One Health approach to date.

The FAO, WHO, and OIE have played a strong role in promoting intersectoral coordination and collaboration at the country level, particularly in Ghana, Sierra Leone, and Tanzania through One Health intersectoral groups and committees [42]. The Fleming Fund also signed an MoU with relevant ministries, departments, and agencies (MDAs) to implement a One Health approach to respond to public health threats through health promotion, early detection, timely response, and post-outbreak management [43].

#### 3.12.2. Raising Awareness of AMR in The Animal Health Sector

The FAO’s awareness-raising activities have targeted a range of stakeholders from food producers and medicine sellers to veterinarians and para-veterinarians in some of the countries included in the review [42]. However, despite the evidence of nationwide, government-supported AMR awareness campaigns in some countries [41], there is a need for more awareness targeting the animal, food, and environmental health sectors in all eight countries.

#### 3.12.3. Antimicrobial Use and Stewardship in Farms

Antibiotics are used in the animal health sector for the treatment and prevention of infections, among other purposes. In addition, they are used in sub-therapeutic doses in livestock feeds to enhance growth and improve feed efficiency in intensive livestock farming [44]. The situation on AMU/AMS [17,41,42,45,46,47,48,49,50,51,52,53,54] across the agricultural sector in the eight countries are depicted in Table S2.

#### 3.12.4. Veterinarians and AMS

In Ghana, there are agencies responsible for monitoring the use of antibiotics and surveillance of resistance in animals. Ad hoc AMR training courses are available for veterinary-related professionals in Ghana, Nigeria, and Sierra Leone [41]. In addition, plans to strengthen the capacity gaps in veterinary services are currently being developed in Sierra Leone [41].

Continued professional training on AMR/AMU exists for veterinary-related professionals in Tanzania and Zambia [41]. In Sierra Leone, the monitoring of veterinary services performance is carried out regularly, e.g., through Performance of Veterinary Services (PVS) Evaluation follow-up missions [41]. No regular training is provided to farmers in Nigeria because it is challenging to engage with them easily. In Malawi, the development of AMS guidelines in the animal sector is still underway.

#### 3.12.5. Antimicrobial Surveillance in Animal and Environmental Sectors

In Kenya, all laboratories performing antimicrobial sensitivity testing are integrated into the AMR surveillance system [41]. Some AMR data are collected locally and fed into the national surveillance system for AMR in livestock and aquaculture.

In the other countries, there are national surveillance systems for AMR in the animal, food, and environmental health sectors. The surveillance plans for AMR in animal health sectors have been approved in Kenya, while the surveillance plans for AMC/AMU in animal health and environmental health sectors are under review in Malawi and Ghana. However, in some countries, the animal health and food safety sectors are not integrated into the national AMR laboratory network. As a result, the antimicrobial sensitivity testing conducted in those labs is not included in the national AMR surveillance system [41]. In Ghana and Tanzania, there are IPC programmes in the animal and plant health services, while in the other countries, there is no evidence of national IPC guidelines covering the animal, food, environment, and agriculture sectors [41].

### 3.13. Stakeholder and AMR Coordinating Committee (AMRCC) Engagement

The eight assessed countries have well established AMRCC committees to facilitate AMR-NAP implementation, with the names of these differing across countries. For example, in Kenya, it is referred to as the ‘National Antimicrobial Stewardship Interagency Committee’ while in Sierra Leone, it is the ‘National Multi-Sectoral Coordinating Group’. The AMRCC oversees and provides overall coordination of the AMR National Action Plan (AMR-NAP) implementation. The activities of the AMRCC are supported by national technical working groups in the implementation of each of the strategic objectives. These technical working groups are composed of technical experts from key stakeholders, representing animal health, food and animal production, human health, the environment, public, private institutions, and civil society.

Currently, the AMRCC in all eight countries has been conducting:

- Assessment of various curricula to determine the extent of inclusion of AMR content;
- Situation analysis of sanitary and phytosanitary measures, IPC, and biosecurity;
- Periodic studies on the efficacy of antimicrobials.

### 3.14. Digital Health

The penetration of mobile phones and other digital technologies has provided an opportunity for digital health initiatives. There has been an increased use of digital health, mirroring global trends fuelled by the COVID-19 pandemic. Additionally, our review demonstrated increased development and implementation of mobile health applications. Countries, such as Ghana, Kenya, Malawi, and Zambia, have adopted national e-Health strategies whilst Nigeria, Sierra Leone, Tanzania, and Uganda are yet to do so [55].

District Health Information Software-2 (DHIS-2), a digital interface designed initially for collecting and using district health information, has been implemented in seven countries. Sierra Leone has not yet implemented the use of DHIS-2 [56].

#### 3.14.1. Advances in the Use of E-Prescribing

Medical apps for healthcare professions have been developed to support adverse event monitoring, prescribing, and quality assurance. These applications are used for various purposes, including point-of-care reference tools, physician consultation, monitoring adherence to therapy, inter-professional interaction, etc.

As part of the CwPAMS programme, the Commonwealth Pharmacists Association (CPA) developed an antimicrobial-prescribing app, to support antimicrobial-prescribing and AMS activities conducted by health partnerships through the programme in Ghana, Tanzania, Uganda, and Zambia by providing guidance, resources, and training documents. The use of apps to aid AMS and inform practice has been well implemented in hospitals [57]. In addition, the UK-led telemedicine initiatives, such as Virtual Doctors have also supported healthcare workers in rural areas in Zambia [58]. However, several barriers to using digital health existed in LMICs, including unavailability of digital tools and connectivity issues [59].

Electronic health services, such as prescribing, are becoming increasingly popular in LMICs. Some examples include the MyDawa app in Kenya [60]; the Surveillance Programme of In-patients and Epidemiology (SPINE) and the MicroGuide app [61,62], implemented in Malawi; and the electronic health records system, ‘Stre@mline’ in Uganda [63].

#### 3.14.2. Use of Medicines Supply Apps or Software

Mobile applications have eased service provision to local hospitals, enabling more efficient and effective distribution of essential medical supplies. However, in some cases, there was no evidence to support whether hospitals had widely implemented the apps [64]. Additional studies on clinical usability and the workflow fit of digital health systems are needed to ensure efficient system implementation. However, this requires support from critical stakeholders, including the government, international donors, and regional health informatics organisations [64].

A study on improving access to antimicrobial prescribing guidelines in four African countries noted initial barriers to uptake that were addressed using a behaviour change approach [59].

### 3.15. Coverage of AMR and AMS in Pharmacy Training

There is a critical shortage of pharmacy personnel, especially in government health centres in all assessed countries. This shortage leads to unqualified personnel managing medicines and supply chains and dispensing medicines to patients, impacting patient care and medicine availability. Countries, such as Kenya, Malawi, Nigeria, Tanzania and Zambia, train pharmacy assistants and technicians for 2–3 years, while pharmacists undergo higher education by completing a 5–6 year Bachelor or Doctor of Pharmacy, respectively. Both the pharmacy technician and pharmacist qualifications require a pre-registration placement [65]. Most of the eight countries have some form of continuing professional development (CPD), designed to update pharmacists’ knowledge and enable them to keep abreast of advancements in pharmaceutical development and modern trends in pharmacy [66]. The uptake of CPD is currently low in countries where it is not mandated, requiring personal motivation to engage [67,68]. The number and role of pharmacists in the eight countries are detailed further in the Table S3.

### 3.16. Access to Antimicrobials, Supply of Medicines

The CwPAMS programme running from 2018 to 2021 highlighted that, in some cases, medicine supply issues led to challenges in achieving AMS. Many African countries are regularly impacted by shortages, substandard/falsified medicines, and supply issues due to COVID-19 and heavy import dependency, especially in the pharmaceutical sector [1,69,70,71,72].

The medicine supply chains of the eight countries in focus have commonalities and variables between them, relating to factors, such as procurement agency type and authority in place. All eight countries procure, source funding from, and distribute essential medicines from a combination of government (Ministries of Health), non-government/not-for-profit organisations, and private sectors. The systems in place are categorised as tier systems, single agency, or devolved.

### 3.17. Access to Medicines via Community Pharmacy

Several studies [17,59] have documented the over-the-counter supply of antibiotics in community pharmacies and enable patient self-medication and misuse of antibiotics. The additional drivers of misuse [73,74] in community pharmacies are highlighted in Box 2

Box 2 **Key drivers of antimicrobial misuse in community pharmacies [73,74].**

Further findings that highlight the key drivers of antimicrobial misuse in community pharmacies include;

- Poor and inefficient public health systems;
- Lack of policies nor strict enforcement;
- Dispensing of antimicrobials in partial doses;
- Sub-optimal AMR knowledge and attitudes among pharmacy personnel;
- Lack of antimicrobial sensitivity knowledge in patients and clients.

### 3.18. Impact of COVID-19

Understandably, COVID-19 has radically shifted the focus of public health agendas. It is, therefore, important to consider how COVID-19 might be impacting the emergence and spread of drug-resistant pathogens through the overuse of medication for the treatment of COVID-19 and secondary infections. COVID-19 has affected the assessed countries’ strides in tackling AMR. The eight countries included in this review operate with several COVID-19 guidelines, and IPC measures are included in these as an integral part of the clinical management of patients. The guidelines highlight the need to ensure standard precautions in all areas of healthcare facilities, including hand hygiene and the use of PPE to avoid direct contact with patients. None of the guidelines mention AMR.

COVID-19 also diverted personnel and resources away from priority diseases, such as HIV/AIDS, TB, malaria, and routine healthcare services, such as antenatal care and vaccination programmes. The full impact of this is yet to be realized.

## 4. Conclusions

This scoping review provides a current overview and assessment of national AMS activities, including IPC programmes and One Health approaches, by eight countries in sub-Saharan Africa: Ghana, Kenya, Malawi, Nigeria, Sierra Leone, Tanzania, Uganda, and Zambia. Notably, all countries have existing NAPs that were developed with the inclusion of pharmacists and reflect the prominence of AMR on the political agenda. Our review further highlighted a significant number of IPC and WASH activities in the eight countries and a notable increase in digital health approaches. However, we also identified a number of shortcomings in national AMS activities, including a lack of documented evidence on AMR and AMU surveillance, AMS activities, and evaluation of NAP implementation. Additionally, there is a need to match increased political motivation to tackle AMR with sufficient investment in technical workforce capacity and expertise in spite of competing healthcare agendas (e.g., around the management of COVID-19). Below, we suggest adaptable recommendations to improve existing AMS practices and increase the sustainability of interventions in the eight countries that were part of this study and beyond.

## 5. Recommendations

The following recommendations for promoting AMS activities are based on the scoping review and focus group discussions with local and national stakeholders in the eight focus countries. They should be adapted to ensure relevance for context and take into account available resources, barriers, and facilitators.

- Develop national AMS plans or guideline documents to facilitate the implementation of national action plans on AMR.
- Increase the number of AMS programmes in healthcare facilities through national roll-outs of successful pilot programmes.
- Incorporate AMS in the curricula for pre-service and in-service training of pharmacists and other healthcare professionals in the national health budget to increase human resources for the in-country implementation of national AMR programmes.
- Identify National Action Plan indicators that need to be improved or upgraded.
- Increase capacity and recognition of technical working groups on AMS.
- Provide technical support to increase the workforce and streamline processes to improve monitoring and evaluation (M&E) for NAPs (Most countries did not have enough literature on the status of AMS implementation).
- Update hospital/healthcare clinical guidelines to include AMS principles and integration of the Access, Watch and Reserve (AWaRe) classification of antibiotics. These customisable guidelines can also be incorporated into existing digital and e-health applications, such as DHIS-2 to optimise prescriptions. The shift towards digitisation can potentially improve antimicrobial prescribing through easy access to information and records.
- Improvement and enforcement of regulations regarding sales of prescription-only antibiotics (There were notable gaps in the policies that govern the sale of antibiotics, especially in community pharmacies. It is important to harness advances in digital technology where possible).
- Streamlining and strengthening national medicine supply chain systems to ensure the consistent availability of quality-assured antimicrobials.
- Encouraging more policies and government regulatory frameworks on the distribution and manufacturing of quality-assured health products at the national level.
- Incorporate bottleneck analysis of national supply chains to identify root causes and human behaviour influencing them.
- The establishment of AMS programmes that incorporate medical supply chain management to facilitate rational antimicrobial selection, procurement, use, monitoring, storage, distribution, and supply.
- The improvement of national quality control and assurance systems of pharmaceuticals to reduce the prevalence of substandard and falsified antimicrobials within the country’s distribution systems and channels.
- The provision of incentives to support AMS programmes and further inclusion/collaboration into/with IPC, WASH, TB and other health programmes in all healthcare facilities.
- The prioritisation of institutionalising and subsequent decentralisation of One Health activities to the sub-national level for implementation, fostering operation, and capacity building.
- Expanding national AMU and AMR surveillance programmes and AMR/AMS awareness campaigns targeted at the stakeholders and MDAs in the animal, food processing, agriculture, and environmental health sectors to enhance coverage.
- The involvement of pharmacists and pharmacy associations further in the implementation of national AMS activities.
- At every opportunity, strengthen and foster partnerships and opportunities that ensure the inclusion of all relevant stakeholders/actors (nationally and internationally) in the implementation of AMS activities.
- The encouragement of more AMR and/or One Health-driven research collaborations between government and non-government stakeholders, with the aim of enhancing the implementation of NAPs.
- Conduct a comprehensive ethnographic study on the use and misuse of antimicrobial drugs in human and animal health, agriculture, and the environment to adequately inform antimicrobial stewardship initiatives.
- Use of digital health tools to support AMS. In Kenya, Sierra Leone, Zambia, and Malawi, it was noted that there is a need for easy access to prescribing information through an app.

## Figures and Tables

**Table 1:** Summary of range of WHO tools used by the focus countries.

	Enrolment to GLASS-AMR	WHO Joint External Evaluation (JEE)	WHO Methodology for PPS	National Action Plans	WHO WASH FIT	WHO Global Plan on AMR	Global TrACSS
Ghana	x	x		x		x	x
Kenya	x	x	x	x	x	x	x
Malawi	x	x	x	x	x	x	
Nigeria	x	x		x	x		x
Sierra Leone		x		x		x	x
Tanzania	x	x		x		x	x
Uganda	x	x	x	x	x	x	
Zambia	x	x		x	x		x

## Supplementary Materials

The following are available online at https://www.mdpi.com/article/10.3390/antibiotics11091149/s1, Box S1: Keywords/Search Terms Box S2: Recommended priority actions for AMR, Box S3: AMR drivers, Box S4: Country-specific examples of AMU, Table S1: Workshops, Key Stakeholders, and Personal Interview Schedule, Table S2: The situation on AMU/AMS in the eight countries for agriculture and animal health, Table S3: The number and role of pharmacists in the eight countries.
