# Supplementary material for: Scoping Review of National Antimicrobial Stewardship Activities in Eight African Countries and Adaptable Recommendations
Source: Antibiotics (Basel). 2022 Aug 25;11(9):1149. doi: 10.3390/antibiotics11091149 (PMC9495160; doi:10.3390/antibiotics11091149)
Supplement: Supplementary file 1 [file antibiotics-11-01149-s001.zip › antibiotics-1841593-supplementary.pdf]

antibiotics

Supplementary material

Scoping Review of National Antimicrobial Stewardship Activities in Eight African Countries and Adaptable Recommendations.

Nduta Kamere [1], Sandra Tafadzwa Garwe [1], Oluwatosin Olugbenga Akinwotu [1], Chloe Tuck [1], Eva M. Krockow [2], Sara Yadav [1], Agbaje Ganiyu Olawale [1], Ayobami Hassan Diyaolu [1], Derick Munkombwe [1,3], Eric Muringu [1,4], Eva Prosper Muro [1,5], Felix Kaminyoghe [1,6], Hameedat Taiye Ayotunde [1], Love Omoniyei [1], Mashood Oluku Lawal [1,7], Shuwary Hughric Adekule Barlatt [1,8], Tumaini J. Makole [1], Winnie Nambatya [1,9], Yvonne Esseku [1,10], Victoria Rutter [1] and Diane Ashiru-Oredope [1,*]

Supplementary Box S1: Keywords / Search Terms

Antimicrobial Resistance, Commonwealth Partnerships for Antimicrobial Stewardship (CwPAMS), Antimicrobial Stewardship (AMS), Antimicrobial Surveillance, National Action Plans (NAP), Infection Prevention and Control (IPC), One Health Strategic Plans, Sub-Saharan Africa (SSA), AMR awareness in Africa farms, Antimicrobial Use (AMU), Antimicrobial Consumption, Antimicrobial Prescription, Antimicrobial Access, AMS Guidelines, Point Prevalence Survey (PPS), Antimicrobial Education, Antimicrobial Regulation, Healthcare-Associated Infections (HCAIs), Antimicrobial Stockouts, Environment, Food, Agricultural, Human and Animal Health Sectors, Water, Sanitation and Hygiene (WASH), Healthcare Waste Management, One Health Policy, One Health Approach, Ghana, Kenya, Malawi, Nigeria, Sierra Leone, Tanzania, Uganda, Zambia

Supplementary Box S2: Recommended Priority Actions for AMR [9-13]

- Developing national action plans to address antimicrobial resistance. This should align with the Global Action Plan on AMR, incorporating action by all relevant sectors, particularly from health, veterinary and agriculture sectors;
- Increasing political engagement, awareness within the health system and actions on AMR;
- Improving systems for surveillance and reporting AMR, including the animal health sector;
- Strengthening laboratory capacity through the involvement in WHO/National Institute for Communicable Diseases (NICD) external quality Assessment (EQA) programme;
- Developing and implementing AMS programmes in health care settings and pharmacovigilance systems in public and animal health sectors;
- Developing and implementing an antimicrobial policy to guide the appropriate prescription and use of antimicrobials in human and animal health;
- Developing Standard Operating Procedures and tools for effective coordination and communication, including AMR data sharing and reporting for human and animal health;

- Harmonising the country's different programmes with different IPC policies and providing information on availability and implementation of IPC at hospitals and the number of trained IPC professionals;
- Improving human resources and infrastructure to boost laboratory capacity for AMR testing for both human and animal health;
- Strengthening quality management systems in the laboratories performing antibacterial susceptibility testing in the human and animal health sectors;
- Improving awareness of the importance of appropriate use of antimicrobials among health care workers, the veterinary workforce, and the public.

Supplementary Box S3 : AMR Drivers [7,15]

- Inadequate Infection Prevention and Control (IPC)/Water Sanitation and Hygiene (WASH) infrastructure in Healthcare Facilities (HCFs) i.e., soap and handwashing;
- Self-medication (patients access the hospital after self-prescribing and there are strong public opinions on antibiotics; Patients have prior knowledge of previous infection treatments, which may lead to the wrong choice of medication, for example, the prevalent use of antibiotics for viral infections);
- Use of antibiotics as a preventative measure;
- Failure for patients to complete their courses of prescribed antibiotics;
- Poor immunisation strategies;
- Low availability of healthcare workers against the patient ratios results in ineffective prescribing;
- Poor knowledge and education amongst antibiotic prescribers;
- Ineffective medicine regulation (over the counter supply and ineffective regulation of supply chains);
- Availability of antibiotics over the counter (clients request for antibiotics);
- Availability of poor quality and or counterfeit antimicrobial agents;
- Misuse of antimicrobial agents in humans, animals, and agriculture;
- Absence/lack of capacity in laboratories to accurately identify and detect resistance. There are few health facilities with lab capacity to do culture and sensitivities across all countries and they face several challenges including lack of reagents, lack of patience by clinicians to wait for lab results before prescribing, long turnaround times for results to come, poor relationships between laboratory staff and the clinicians and poor teamwork);
- The difficulties in differentiating a viral infection with a bacterial one makes prescribers make unnecessary antibiotic prescriptions for even viral infections;
- Lack of proper surveillance programmes for Multidrug-Resistant Organisms at the local and national level;
- Subsidising the cost of medicines through health insurance has been shown to increase the consumption of medicines especially antibiotics, which in turn may contribute to irrational use;
- Poor documentation and poor compliance with treatment guidelines are common problems encountered in antimicrobial prescribing.

Supplementary Box S4 : Country-specific examples of AMU activities [24-28]

According to the in-country consultant in Uganda, at all AMR designated surveillance sites, there has been rejuvenation of Medicine and Therapeutics Committees (MTCs), boosting IPC capacity, Point Prevalence Surveys (PPS) and application of WHO AWARE tool to address antimicrobial consumption (AMC) challenges. There are also ongoing efforts to set up the Pharmacy AMR National Coordination Centre (NCC) at the Ministry of Health (MoH) pharmacy dept and share data with GLASS. In Malawi, sentinel sites are used to monitor AMR for TB, paediatric meningitis, rotavirus and HIV.

The workshops showed that WHO PPS Survey has been done in local hospitals in Sierra Leone. Data has been collected but has not yet been published. In addition, a PPS was performed in the Kilimanjaro Christian Medical Centre (KCMC) (tertiary hospital), Mawenzi (regional) and St. Joseph (district) hospital in Tanzania from November and December 2016. Antibiotic use in all patients admitted for more than 24 hours and those undergoing surgery was recorded. The results of the PPS in these three hospitals from different levels of healthcare in Tanzania showed a prevalence of antibiotic use in hospital patients of 44%. This is comparable to results from African countries that have participated in the Global PPS (50%) and surveys from individual sub-Saharan African countries such as Botswana 70.6% and Kenya 67.7%.

In Kenya, in-country consultants indicated that a retrospective review of antimicrobial consumption had been conducted with the support of the Fleming Fund through IQVIA, data analysis and report writing are in progress. In addition, PPS in 6 hospitals have been finalised and data analysis and report writing are ongoing.

Malawi has approximately 40% of hospital laboratories detecting, isolating and identifying antimicrobial-resistant organisms. Reports, however, show insufficient action on several antibiotic sensitivity results. Currently, sentinel sites are used for AMR surveillance monitoring for TB. According to the in-country consultant, the surveillance programme is not done at a national level but is more efficient at the institutional level through partnerships and collaboration. The national microbiology reference laboratory (NMRL) monitors nine priority pathogens from human and veterinary diagnostic laboratories. Standard treatment guidelines are under review using NAP and GLASS routine data. Based on the focus group discussions, PPS is underway in 7 Fleming Fund health facilities out of a total of 34. Albeit being delayed due to COVID-19 and ethical issues, there are plans to expand. As per requirement, there was a need for ethical approval from the National Ethics Committee if findings are to be published. The data will be used to inform AMR guidelines. However, PPS is needed in other facilities, which is currently a challenge due to a lack of funds.

Nigeria was part of the African countries trained on the WHO methodology for PPS with Botswana, Burkina Faso, Burundi, Cameroon, Côte d'Ivoire, Kenya, Madagascar, Mozambique, United Republic of Tanzania, and Zimbabwe. The training allowed for the development of operational plans for conducting national surveys by 2019. As a result, the country has adopted the WHO PPS tool, which provides for reporting stockouts.

Supplementary Table S1 : Workshops, Key Stakeholders and Personal Interview Schedule

Uganda and Malawi
Thursday 1st April, 10am-12pm, UK time (BST)

Invited Participants:

Role/Area of representation	Uganda	Malawi
In-Country Consultant	Winnie Nambatya	Felix Kaminyoghe
National AMR Committee	Mr Ibrahim Mugerwa, Secretary to the National AMR committee	Dr Watipaso Kasambala, Country AMS Coordinator
One Health	Mr Musa Sekamate, Coordinator for the One Health Office	Dr Peter Mwale, One Health Coordinator
AMS Lead	Ms Akello Zainab, Lead for AMS CwPAMs project in Gulu Regional Referral Hospital	Dr Titha Dzowela, Member of AMS team
IPC Lead	Ms Akello Stella, Focal Person for IPC in Gulu Regional Referral Hospital	Dr Owen Msopole, IPC Coordinator
Doctor	Mr Okot Christopher, Doctor and Member of the AMS sub-committee	
Nursing	Ms Akello Beatrice, Nurse and Member of the AMS sub-committee	
Hospital Pharmacy	Olum William, Hospital Pharmacist, Jinja Regional Referral Hospital	Chikhulupiliro Chimwaza, AMS Pharmacist<br>Sam Nowa, Hospital Chief Pharmacist
		Dr Isaac Shawa, Diagnostics Representative<br>Confidence Banda, Medical Laboratory UNION President

Kenya
Thursday 1st April, 1pm-3pm, UK time (BST)

Invited Participants:

Role/Area of representation	Kenya
In-Country Consultant	Eric Muringu
National AMR Committee	Dr. Evelyn Wesangula, Head, Patient Safety, MOH

One Health	Dr. Allan Azegele, MR focal point person at the Directorate of Veterinary Services
AMS Lead	Dr. Collins Jaguga, Technical Advisor, MTaPS<br>Dr. Kusu Ndinda, Country Director MSH, MTaPS
IPC Lead	Veronica Kamau, IPC, MOH
Doctor	Dr. Irungu Kamau, MOH
Nursing	Christopher Kibiwott, Nurse, Kenyatta National Hospital
Hospital Pharmacy	Dr. Susan Mutua, Chief Pharmacist, Gertrude's Children's Hospital

Ghana
Thursday 22nd April, 10am-12pm, UK time (BST)

Invited Participants:

Role/Area of representation	Ghana
In-Country Consultant	Yvonne Yirenkyiwaa Esseku
National AMR Committee	Dr Martha Gyansa-Lutterodt<br>Joycelyn Azeez
One Health	Saviour Yevutse
AMS Lead	Kwame Ohene Buabeng
IPC Lead	Mary Ayram Ashinyoh
Doctor	Mary Ayram Ashinyoh
Nursing	Yaa Obirikorang
Hospital Pharmacy	Daniel Ankrah

Zambia and Nigeria
Thursday 22nd April, 1pm-3pm, UK time (BST)

Invited Participants:

Role/Area of representation	Zambia	Nigeria
In-Country Consultant	Dr Derick Munkombwe	Mashood Lawal

National AMR Committee	Dr Kaunda Yamba	Dr Tochi Okwor
One Health	Dr Otridah Kapona	Dr Abiodun Egwuenu
AMS Lead	Ms Uchizi Chola Chirwa	Prof Oladipo Aboderin
IPC Lead	Mr Luke Alutuli	Dr Tochi Okwor (as above)
Doctor	Dr Sydney Shampile	Dr Babatunde Ogunbosi
Nursing	Sr Emily Muyoma	Ayoola Modupe Christiana
Hospital Pharmacy	Mr Divine Mbambala	Ibikunle Abosede Esther
Pharmacy		Gbenga Joseph

Tanzania
Thursday 29th April, 1pm-2.30pm, UK time (BST)

Invited Participants:

Role/Area of representation	Tanzania
In-Country Consultant	Eva Prosper Muro
National AMR Committee	Dr. Doreen Mloka<br>Rose Shija
One Health	Dr. Judy Jubilate
AMS Lead	Siana Mapunjo
IPC Lead	Dr. Joseph Hokororo
Doctor	Dr. Furaha Lyamuya
Nursing	No representative
Pharmacy	Daudi Msasi<br>Hamidu Rajabu<br>Mary Kisima

Sierra Leone
Thursday 6th May, 11am, UK time (BST)

Invited Participants:

Role/Area of representation	Sierra Leone

In-Country Consultant	Dr Shuwary HA Barlatt
National AMR Committee	Dr Kanu Joseph
One Health	Mr Bunting Graden
AMS Lead	Pharm Janet Buck
IPC Lead	Mrs Christiana Kallon
Doctor	Sulaiman Lakoh
Nursing	Mrs Aminata Koroma
Hospital Pharmacy	Pharm Brima Lahai

Supplementary Table S2 : The situation on AMU/AMS in the eight countries for agriculture and animal health [17],[41],[42],[45-54]

**Ghana**	In research conducted in urban Ghana, farmers use antibiotics for disease prevention. Laws that prohibit the use of antibiotics for growth promotion are under development as well as guidelines on prescribing antimicrobials.
**Kenya**	Managers or farmers are the key decision makers with about 70% of the farms using antibiotics. Recently, a plan has been agreed upon for monitoring quantities of antimicrobials sold for/used in animals, based on OIE standard. Tailored ad hoc AMR training courses are available for at least two groups of OH key stakeholders.
**Malawi**	In this country, farmers are reportedly facing a lack of regular and coordinated drug supply. There are also national treatment guidelines, but no mechanisms to ensure or enforce appropriate prescription and use of antimicrobials in animal health and food production.
**Nigeria**	There is no legislation on sales and prescription of antimicrobial medicines for animal use, nor are there laws that prohibit the use of antibiotics for growth promotion in the absence of risk analysis despite the issued ban by the NAFDAC. Tetracyclines are the most commonly misused antibiotics in Nigerian farms. Meanwhile, data is collected and reported on AMU/AMC in the animal sector.
**Sierra Leone**	There is lack of regulation on AMS in the animal and environmental sectors, which may be due to the absence of national policy or legislation in optimising AMU regarding the quality, safety and efficacy of antimicrobial products, and their distribution in animal health sector . There are no training provisions on AMR/AMS for

	key stakeholders such as farmers (though ad hoc AMR courses are available for veterinary related professionals).
**Tanzania**	About 100% of the farms are using antimicrobials in animal production. On a regular basis, data is collected and reported to the OIE on the total quantity of antimicrobials sold for/used in animals. There are no training provisions on AMR/AMS for OH key stakeholders.
**Uganda**	The NDA keeps records of all antimicrobials imported into the country and periodically collates them, but they are not currently widely shared. The AMR/AMS guidelines for the animal, food processing and safety, agriculture, fisheries and environment sectors are still under review by the Ministry of Agriculture, Animal Industries and Fisheries (MAAIF).
**Zambia**	In Zambia, almost all the farms use antimicrobial agents based on farmers' knowledge and experiences. The country now has laws on the prescription and sale of antimicrobials for animal use, while regulations that prohibit the use of antibiotics for growth promotion in the absence of risk analysis are yet to be developed.

Supplementary Table S3 : The number and role of pharmacists in the eight countries

Country	Estimated number of pharmacists	Estimated number of pharmacy technicians	Main sectors of pharmacists
Ghana	5098	2298	Clinical, community, education, industry, pharmaceutical marketing, public health, social and administrative pharmacy
Kenya	4,000	11,000	Public sector – government hospitals, community pharmacists, industry, manufacturing, importation, distribution and wholesale, academia
Malawi	200*	Not available	Information not available
Nigeria	29,589	9,172	Dispensing, counselling, clinical ward rounds, members of medicines/ thera-

			peutic committees, supply chain management, AMS, traditional medicines (herbal), administration
Sierra Leone	203	364	Supply chain activities (district and central level), Clinical (in some hospitals) as part of the clinical team, as part of drug and therapeutics committees, regulatory affairs, community pharmacies
Tanzania	2599	3525 and 746 pharmaceutical assistants enlisted	Hospital settings: Hospital formulary development, Ethical dispensing, Inventory management, Medication reconciliation, Adherence counselling, Compounding of Extemporaneous preparations, Adjustment of Prescriptions, Antimicrobial Stewardship lead person, documentation and record keeping in dispensing, Dispensing, counselling, clinical ward rounds, Secretary and members of medicines/ therapeutic committees, Supply chain activities in procurement and store management, medicines distribution Non-hospital settings – Academia in Schools of Pharmacy, Regulatory institution Tanzania Medicines and Medical Devices Authority (TMDA), Community pharmacies and Medical stores
Uganda	1445	Not available	Information not available
Zambia	1513	2067	Dispensing medicines, attending clinical rounds, procurement and store management, medicines distribution. Non-hospital settings - Academia, regulatory institutions, community pharmacies and medical stores
